# *COL1A2* expression is associated with prognosis, stromal remodeling, and predicted immunotherapy response in gastric cancer

**DOI:** 10.3389/fonc.2026.1912517

**Published:** 2026-10-30

**Authors:** Fengming Luan, Guofeng Chen, Ke Chen, Chaoyang Zhang, Xiaoman He, Jian Chen, Shenbin Xu

**Affiliations:** Department of Gastrointestinal Surgery, The Second Affiliated Hospital of Zhejiang University School of Medicine, Hangzhou, Zhejiang, China

**Keywords:** COL1A2, extracellular matrix, gastric cancer, immunophenoscore, prognosis, tide

## Abstract

**Background:**

COL1A2 encodes type I collagen alpha2 chain, a stromal component in gastric cancer (GC); its prognostic value, cellular origin, and immune microenvironment relation remain unclear.

**Methods:**

We analyzed TCGA-STAD, GSE63089, and ACRG GSE62254. Associations between COL1A2 abundance and overall survival (OS), clinicopathology, pathways, and tumor microenvironment were assessed. High/low groups by median expression. Two Cox models used pathological stage or separate T/N/M. Immunotherapy response predicted via TIDE and IPS. WGCNA, PPI, and LASSO with five ECM genes, ten-fold cross-validation and 200 repeats, were applied. qPCR and Western blot measured mRNA and protein in seven paired tissues.

**Results:**

COL1A2 was higher in GC and linked to poorer OS, but time-dependent AUCs were 0.60, 0.65, 0.66. In ACRG, OS association was not significant (HR 1.83, P = 0.085). Per SD increase, adjusted HR was 1.21 (stage model, P = 0.036) and 1.14 (T/N/M model, P = 0.171). C-index gains were only 0.006 and 0.003. Higher abundance correlated with stroma-rich microenvironment and M2 macrophage infiltration. TIDE/IPS predicted lower ICI response probability. LASSO selected SPARC, ELN, and BGN stably. Tumor tissues showed higher mRNA and protein.

**Conclusions:**

COL1A2 associates with poor prognosis, stromal remodeling, and predicted immunotherapy response, but its prognostic effect varies between models and discrimination improvement is small.

## Highlights

- COL1A2 encodes the alpha2 chain of type I collagen, a central component of the GC extracellular matrix. Its transcript abundance was higher in GC tissues and was associated with poorer survival in TCGA-STAD, a stroma-rich microenvironment, and M2 macrophage estimates. The external ACRG GSE62254 analysis of 300 matched tumors was not significant (HR 1.83, P = 0.085; log-rank P = 0.120). Its adjusted hazard ratio was 1.21 in the pathological-stage model but 1.14 and nonsignificant in the separate T/N/M model, and incremental C-index gains were below 0.01. GSE63089 validated expression but not survival. Higher TIDE and lower IPS values indicated less favorable model-based, not clinically observed, ICI response scores. Repeated LASSO cross-validation showed high selection stability for SPARC, ELN, and BGN among the exploratory network genes, and the seven-pair series confirmed increased bulk-tissue COL1A2 mRNA and protein abundance.

## 1 Introduction

Gastric cancer (GC) is one of the most prevalent malignancies globally, with over 1,000,000 new cases and approximately 769,000 deaths annually, making it the fifth most prevalent cancer and the fourth leading cause of cancer-related mortality worldwide (1). While the five-year survival rate for early-stage diagnoses can reach up to 70%, it drops below 30% in cases involving invasive disease or distant metastasis (2). Unfortunately, many patients are diagnosed at advanced stages, when curative surgical options are no longer viable (3). Stomach adenocarcinoma (STAD) represents nearly 90% of all GC cases. Due to the absence of distinct clinical symptoms in the early stages, 80–90% of patients with STAD are diagnosed only after the disease has reached an advanced stage (4). Consequently, the identification of reliable biomarkers for early detection and timely therapeutic intervention is critical to improving GC prognosis.

The extracellular matrix (ECM) is a multifaceted network of fibrous proteins, proteoglycans, and various signaling molecules, including cytokines, growth factors, and hormones (5). As a central regulator of cellular and tissue homeostasis, the ECM plays a pivotal role in maintaining physiological function. In cancer, the ECM constitutes a vital component of the tumor microenvironment, where it governs several tumor cell behaviors, such as proliferation, migration, invasion, and differentiation, thus promoting tumor progression (6). Collagen (COL), the predominant fibrous protein in the ECM, is integral to cancer biology (7). COL consists of polypeptide α-chains that assemble into homo- or heterotrimers, with 28 distinct COL types identified to date (8). Enhanced COL cross-linking and deposition are linked to the activation of integrin signaling pathways, which subsequently stimulate tumor growth (9). COL genes also play a role in ECM–receptor interactions and focal adhesion pathways, both essential for tumor invasion and metastasis (10). Guszczyn and Sobolewski observed increased COL turnover in GC tissues, which may contribute to ECM disorganization (11). Tumor samples from patients with GC exhibited elevated levels of type I and IV COL, along with fibronectin and laminin, when compared to normal tissues (12). Type I COL (COL I), the most abundant ECM component in organs such as the pancreas (13), is a heterotrimer composed of two α1 chains (encoded by *COL1A1*) and one α2 chain (encoded by *COL1A2*) (14).

The *COL1A2* gene has been extensively studied as a model to explore the molecular mechanisms of COL I biosynthesis (15). Recent research has highlighted *COL1A2* as a pivotal gene in colorectal cancer, where it plays a critical role in tumor progression and acts as a prognostic marker for stage IIA colon cancer (16). In glioblastoma, *COL1A2* inhibition has been shown to suppress tumor proliferation by inducing cell cycle arrest (17). Additionally, *COL1A2* serves as a regulatory gene in pancreatic cancer (18), inhibiting cell proliferation, migration, and invasion in colorectal (19) and bladder (20) cancer cells, while reducing colony formation capacity in hepatic epithelial cells *in vitro* (21). Collectively, these findings suggest that *COL1A2* may function as a tumor suppressor or regulatory factor across various cancer types, with its activity closely associated with cancer development and progression.

Nevertheless, the biological and clinical significance of COL1A2 in GC remains incompletely understood, particularly in relation to stromal composition, immune infiltration, and immunotherapy response. Because COL1A2 encodes the alpha2 chain of type I collagen, it may be relevant to the collagen-rich extracellular matrix and stromal remodeling that characterize GC. We examined its expression pattern, prognostic association, functional context, and immune correlates in public GC datasets, and further evaluated its relationship with co-expressed ECM-related genes.

## 2 Materials and methods

### 2.1 Data acquisition and processing

Processed TCGA-STAD gene-level RNA-seq and clinical matrices were obtained from the UCSC Xena TCGA hub (https://xenabrowser.net/datapages/). The expression matrix contained 415 primary tumors and 35 solid-tissue normal samples and comprised log2-transformed RSEM-normalized values; 412 tumor cases had usable survival information. GEO series GSE63089 comprised 45 paired GC and adjacent normal tissues profiled on GPL5175. GSE62254 (ACRG) expression profiles were obtained from the GEO series matrix (300 gastric tumors), and OS and follow-up status were obtained from the ACRG clinical information table (**Supplementary Data 1**; 22). TIMER2.0 (http://timer.cistrome.org/) was used to place the STAD expression pattern in a pan-cancer context. Human Protein Atlas (HPA; https://www.proteinatlas.org/) images were used only as publicly available, illustrative tissue-level protein examples and were not used for quantitative or semiquantitative comparison. Analyses were performed in R version 4.5.2. The analysis datasets, result tables, and source scripts are provided in this study's **Supplementary Data 1**

### 2.2 *COL1A2* expression and survival analyses

COL1A2 expression was compared between tumor and normal tissues and independently assessed in GEO series GSE63089. For dichotomized analyses in TCGA-STAD, patients were categorized using the median log2 RSEM-normalized COL1A2 value among the 415 primary tumors (14.894): values greater than or equal to the median were classified as high expression and lower values as low expression. This cutoff was prespecified from the expression distribution and was not selected by survival optimization. Kaplan-Meier curves were then used to compare overall survival (OS). For GSE62254, the GPL570 probe 202404_s_at, which had the highest mean signal among probes annotated to COL1A2, was used. GEO sample titles (T followed by the patient number) were matched to the numeric Tumor ID in the ACRG clinical table. Follow-up status codes 0 and 1 were treated as censored observations, and codes 2, 3, and 4 as deaths. Univariable Cox regression, median-defined Kaplan-Meier curves, and log-rank testing were performed in all 300 matched tumors; the GSE62254 median COL1A2 value was 3.691 RMA-normalized units.

### 2.3 Time-dependent ROC and clinical correlation analyses

Time-dependent ROC curves were used to summarize 1-, 3-, and 5-year OS discrimination by *COL1A2* expression. Clinicopathological comparisons included age, sex, histological grade, pathological stage, and T, N, and M classifications. For the N/M analysis, N1-N3 were combined and compared with N0, while M1 was compared with M0. Because the Xena expression values are on a log2 scale, between-group mean differences were exponentiated as fold changes and reported with 95% confidence intervals, Hedges’ g, and two-sided P values.

### 2.4 Multivariable Cox regression and model performance

Cox proportional-hazards models were fitted using complete cases. *COL1A2* was standardized so that its hazard ratio represented a one-standard-deviation increase. Model A included age, sex, grade, pathological stage, and *COL1A2*; Model B replaced pathological stage with separate T, N, and M classifications. Proportional-hazards assumptions were evaluated with Schoenfeld residual tests. Harrell C-indices for the clinical models with and without *COL1A2* were compared using 500 bootstrap resamples.

### 2.5 Functional enrichment analysis

Differentially expressed genes (DEGs) between the median-defined high- and low-COL1A2 expression groups were identified using the limma package. A log2 fold change (FC) > 1 and an adjusted P value < 0.05 were considered thresholds for DEG identification. Gene Ontology (GO) functional enrichment analysis and Kyoto Encyclopedia of Genes and Genomes (KEGG) pathway analysis were performed using the “ClusterProfiler” (v4.10.1) R package. Gene set enrichment analysis (GSEA) was conducted with the same package using the c2.cp.kegg.v7.4.symbols.gmt gene set, and an adjusted P value < 0.05 was considered statistically significant.

### 2.6 Immune microenvironment and infiltration analyses

Patients with GC were categorized into median-defined high- and low-COL1A2 expression groups as described above. The R ESTIMATE package was used to calculate stromal, immune, and ESTIMATE scores for each tumor sample from gene-expression profiles (23). Group differences in these scores were assessed between the two expression groups. The R CIBERSORT package was used to estimate immune-cell fractions in bulk tumor tissue. Spearman correlation analysis was used to assess associations between COL1A2 expression and estimated immune infiltration or immune-checkpoint transcripts, with two-sided P < 0.05 considered statistically significant. Because these methods are applied to bulk transcriptomes, the resulting scores and cell fractions were interpreted as compositional estimates rather than direct measurements.

### 2.7 Immunotherapy response scoring

Patients with GC were divided into median-defined high- and low-COL1A2 groups. Immunophenoscore (IPS) values were obtained from The Cancer Immunome Atlas (TCIA; https://tcia.at/), and tumor immune dysfunction and exclusion (TIDE) scores were generated through the TIDE portal (http://tide.dfci.harvard.edu/).

### 2.8 Weighted gene co-expression network analysis

Expression profiles and the IPS and TIDE metrics were used to construct a weighted gene co-expression network with the WGCNA R package. The top 50% of genes retained by the original variability filter formed the input matrix. Pearson correlations were used to construct the network and examine module-trait associations. Modules with nominal P < 0.05 were considered associated with the immune-response scores, and the module with the largest absolute correlation was selected for downstream analysis.

### 2.9 PPI network construction and hub-gene selection

Genes from the WGCNA module of interest were submitted to STRING (https://string-db.org/) to obtain interaction information. The PPI network was visualized in Cytoscape, and densely connected candidate genes were identified with the MCODE algorithm.

### 2.10 LASSO feature selection

LASSO-penalized Cox regression was performed for COL1A2 and the five ECM-related genes retained by the preceding survival screen (SPARC, THBS2, ELN, BGN, and COL5A1). Predictors were standardized. Lambda was selected by ten-fold cross-validation using partial-likelihood deviance, and both lambda.min and the one-standard-error lambda (lambda.1se) were reported. Selection stability was assessed over 200 repeated ten-fold cross-validation runs with master seed 20260729.

### 2.11 Patient samples

Seven patients with gastric cancer treated at the Second Affiliated Hospital of Zhejiang University School of Medicine between March and April 2025 were enrolled. Paired tumor and adjacent non-tumor tissues were collected from surgical specimens and used solely for tissue-level expression validation. Available age, sex, and collection-date records for the seven-patient cohort are summarized in **Supplementary Table 1**. Detailed clinicopathological variables and a standardized recorded distance from the tumor margin were not retained; therefore, this small series was not used for subgroup, clinicopathological, or outcome analyses. The study was approved by the institutional ethics committee (approval No. 20241481), conducted in accordance with the Declaration of Helsinki, and supported by written informed consent from every participant.

### 2.12 Experimental methods and statistical procedures

#### Quantitative real-time PCR

Total RNA was extracted from 20 mg tissue using the FastPure Cell/Tissue Total RNA Isolation Kit V2 (Vazyme, catalog No. RC112-01), and 1 microgram RNA was reverse-transcribed with HiScript III RT SuperMix for qPCR (+gDNA wiper; Vazyme, catalog No. R323-01). Each 20-microliter qPCR reaction contained 10 microliters of 2x ChamQ Universal SYBR qPCR Master Mix (Vazyme, catalog No. Q711-02), 0.4 microliter each of 10-micromolar forward and reverse primers, 4 microliters of cDNA template, and nuclease-free water to volume. The primer sequences were: GAPDH forward, AGAAGGCTGGGGCTCATTTG; GAPDH reverse, AGGGGCCATCCACAGTCTTC; COL1A2 forward, CTGGTAGTCGTGGTGCAAGT; and COL1A2 reverse, AATGTTGCCAGGCTCTCCTC. Amplification was performed on an Applied Biosystems 7500 system with 95 degrees C for 30 seconds, followed by 40 cycles of 95 degrees C for 10 seconds and approximately 60 degrees C for 30 seconds, and a melt curve of 95 degrees C for 15 seconds, 60 degrees C for 60 seconds, and 95 degrees C for 15 seconds. The COL1A2 amplicon was 146 bp. Each sample was assayed in three technical replicates.

Technical replicates were averaged within each biological tissue specimen before statistical analysis. The biological tissue pair, rather than an individual technical replicate, was the statistical unit (n = 7 paired specimens). qPCR relative-expression values and normalized Western blot densitometry values were compared between tumor and matched adjacent tissues using two-sided paired t-tests and are presented as mean +/- standard deviation (SD).

### 2.13 Immunohistochemistry

Immunohistochemistry (IHC) was not used as an analytical validation assay in this revision because the original IHC images, scale calibration, and scoring records could not be retrieved. To avoid unsupported quantitative or localization claims, all **Figure 1** conclusions are based on the traceable paired qPCR and Western blot measurements.

**Figure 1:**
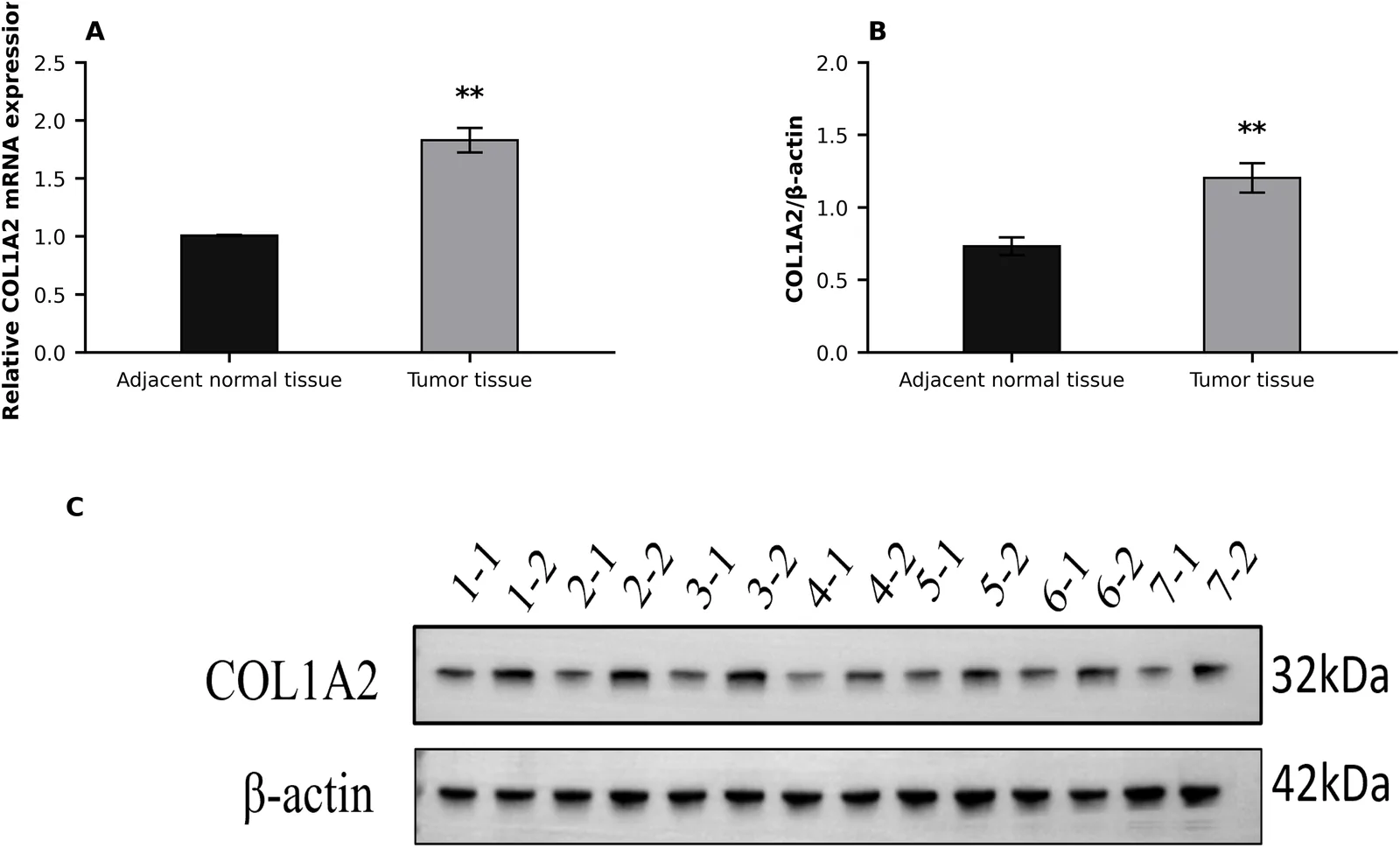
COL1A2 mRNA and COL1A2 protein abundance in seven paired gastric cancer tissues. **(A)** Relative COL1A2 mRNA abundance by qPCR; tumor and matched adjacent tissues were compared using a two-sided paired t-test after technical replicates were averaged within each biological specimen (mean +/- SD: 1.83 +/- 0.28 vs 1.01 +/- 0.01; P < 0.001). **(B)** Densitometric COL1A2/beta-actin protein ratio, analyzed in the same paired manner (mean +/- SD: 1.20 +/- 0.27 vs 0.73 +/- 0.16; P < 0.001). **(C)** Western blot bands for the seven pairs; -1 denotes adjacent normal tissue and -2 denotes matched tumor tissue. **P < 0.01 applies to the paired qPCR and Western blot quantitative comparisons.

### 2.14 Western blotting

Tissue samples were washed with PBS in an ice bath, chopped, and homogenized in RIPA lysis buffer (Beyotime Biotechnology Co., Ltd., Shanghai, China, catalog No. P0013B) containing protease inhibitors using a tissue homogenizer (Scientz-12, Ningbo Scientz Biotechnology Co., Ltd., Ningbo, China) at 70 Hz for 200 seconds. The samples were lysed on ice for 30 minutes and then centrifuged at 11,000g and 4 degrees C for 10 minutes to remove cell debris. The supernatant was collected and stored on ice. Protein concentration was quantified using the BCA protein assay kit (HaoKe Biotechnology Co., Ltd., Hangzhou, China, catalog No. hkw2019). Proteins were resolved by 12% SDS-PAGE and transferred to a PVDF membrane. After blocking with 5% skim milk in TBST for 1 hour, membranes were incubated overnight at 4 degrees C with rabbit polyclonal anti-COL1A2 antibody (Antibody System, catalog No. PHC30201; 1:1000; reported molecular weight 32 kDa) and mouse monoclonal anti-beta-actin antibody (Diagbio Co., Ltd., Hangzhou, China, catalog No. db13986; 1:5000; reported molecular weight 42 kDa). Membranes were then incubated for 1 hour with HRP-conjugated secondary antibodies appropriate to the host species of the primary antibodies. The goat anti-rabbit secondary antibody used for COL1A2 was obtained from MultiSciences (Lianke) Biotechnology Co., Ltd. (Hangzhou, China; catalog No. GAR0072; 1:5000). Target protein expression was detected using an ECL color development kit (Biosharp Biotechnology Co., Ltd., Hefei, China, catalog No. BL520A).

Band intensities from the seven paired tissues were quantified with ImageJ and normalized to beta-actin. Values are presented as mean +/- SD, and tumor and adjacent tissues were analyzed as paired biological samples.

### 2.15 Statistical analysis

Statistical analyses were performed in SPSS version 26.0 and R version 4.5.2. Quantitative data are summarized as mean +/- SD. For the seven paired tissue samples, technical replicates were averaged first, each patient contributed one tumor-adjacent tissue pair, and qPCR relative expression and Western blot densitometry were compared using two-sided paired t-tests. No IHC image or score was subjected to inferential statistical testing. Cox models report hazard ratios with 95% confidence intervals, and proportional-hazards assumptions were assessed with Schoenfeld residuals. The GSE62254 external analysis used univariable Cox regression, median-defined Kaplan-Meier curves, and a two-sided log-rank test. All P values were two-sided, with P < 0.05 considered statistically significant.

## 3 Results

### 3.1 *COL1A2* expression and prognostic association in public datasets

TIMER2.0 pan-cancer visualization placed the STAD expression pattern in the context of other cancer types (**Figure 2A**). TCGA-STAD analysis showed higher COL1A2 transcript abundance in tumor than in normal tissues (**Figure 2B**), and GSE63089 showed the same expression pattern (**Figure 2C**). Patients in the median-defined high-COL1A2 transcript group had poorer OS than those in the low-transcript group (**Figure 2D**). Time-dependent ROC analysis yielded AUC values of 0.60, 0.65, and 0.66 at 1, 3, and 5 years, respectively (**Figure 2E**). The HPA images are included only as illustrative examples of tissue staining and were not used as quantitative evidence of differential protein abundance (**Figures 2F, G**).

**Figure 2:**
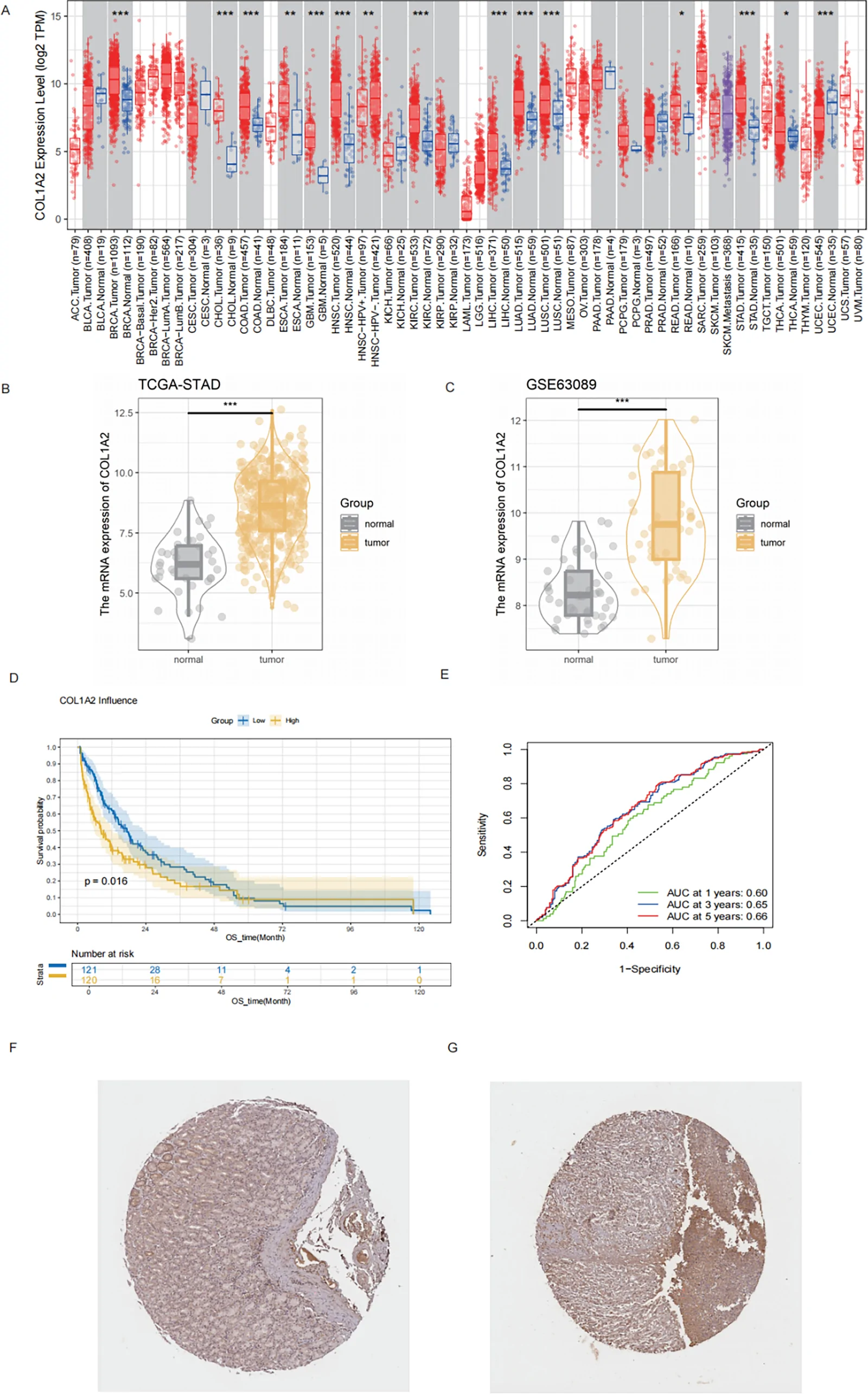
COL1A2 transcript abundance and prognostic association. **(A)** TIMER2.0 pan-cancer visualization, included to contextualize the STAD expression pattern. **(B)** COL1A2 transcript abundance in TCGA-STAD tumor and normal tissues. **(C)** Independent transcript-abundance comparison in GEO series GSE63089. **(D)** Kaplan-Meier overall survival curves for median-defined high- and low-COL1A2 transcript groups (cutoff: 14.894 log2 RSEM-normalized units). **(E)** Time-dependent ROC curves for 1-, 3-, and 5-year overall survival. **(F, G)** Publicly available HPA examples of COL1A2 protein staining in normal stomach and gastric cancer tissues.

External survival validation in GSE62254 included all 300 gastric-tumor profiles with matched OS records, including 152 deaths and 148 censored observations. Using the COL1A2 probe 202404_s_at and the cohort median expression (3.691 RMA-normalized units), higher COL1A2 was not significantly associated with OS in univariable Cox analysis (HR 1.83, 95% CI 1.02-3.30; P = 0.085). Median-defined Kaplan-Meier curves were likewise not significantly different (log-rank P = 0.120; **Supplementary Figure 1**).

### 3.2 Associations between *COL1A2* expression and clinicopathological features

Associations between *COL1A2* expression and clinicopathological features were examined in TCGA-STAD. Higher expression was observed with advanced T classification and pathological stage (**Figures 3A, D**). N1-N3 and N0 had nearly identical mean expression (fold change 0.99, 95% CI 0.81-1.21; Hedges’ g = -0.014; P = 0.898; n = 274 versus 123). M1 showed a small, imprecise elevation relative to M0 (fold change 1.16, 95% CI 0.79-1.69; Hedges’ g = 0.150; P = 0.453; n = 27 versus 367). No significant difference was observed by N or M classification (**Figures 3B**, **C**). Histological grade, sex, and age comparisons are shown in **Figures 3E**–**G**.

**Figure 3:**
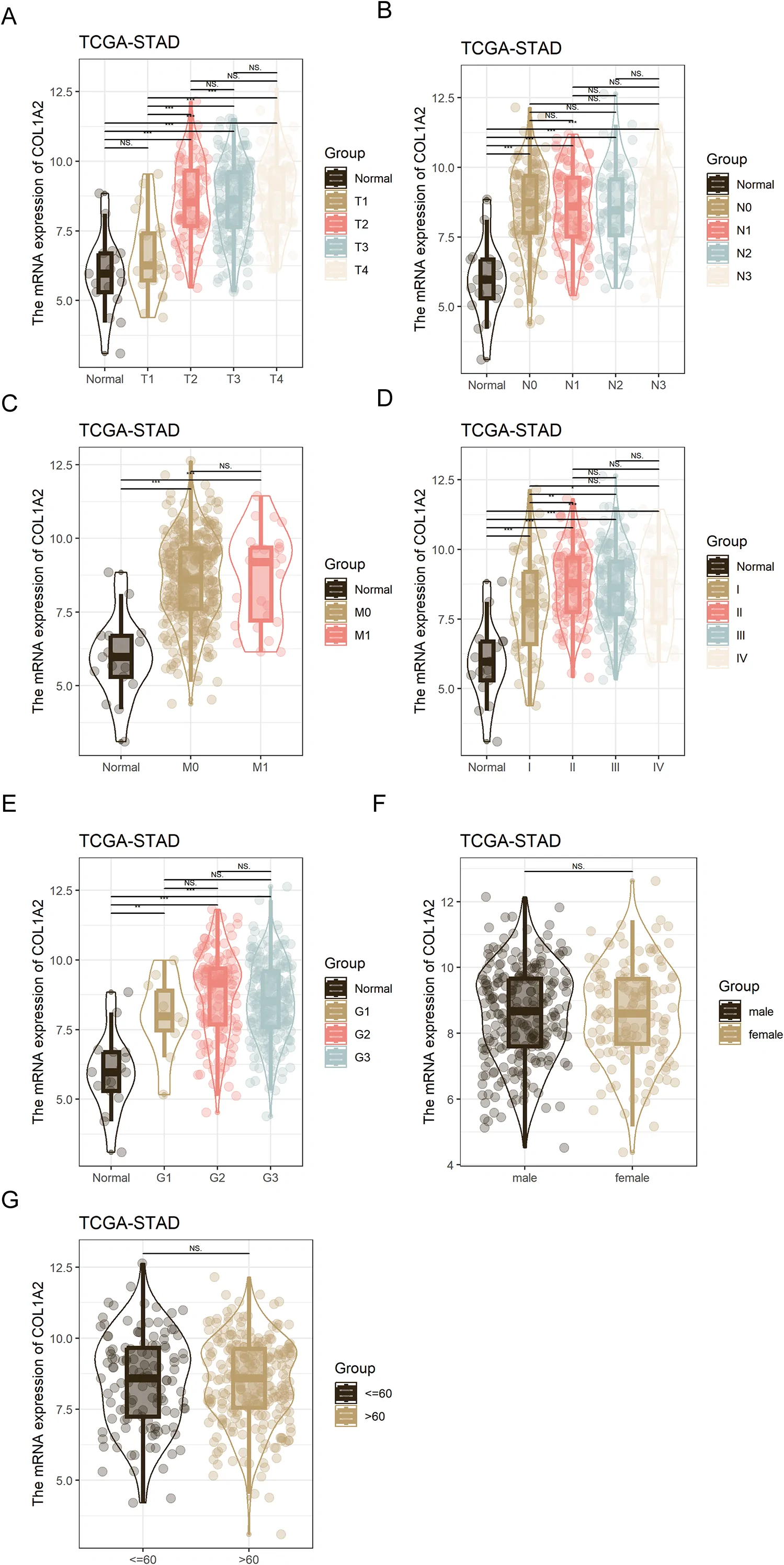
Associations between *COL1A2* expression and clinicopathological features. *COL1A2* expression is shown by **(A)** T classification, **(B)** N classification, **(C)** M classification, **(D)** pathological stage, **(E)** histological grade, **(F)** sex, and **(G)** age group. Nonsignificant comparisons are labeled NS.

### 3.3 Multivariable Cox models and prognostic performance

In the unadjusted analysis of 407 cases, each standard-deviation increase in *COL1A2* was associated with poorer OS (HR 1.25, 95% CI 1.06-1.46; P = 0.006). In Model A, which adjusted for age, sex, grade, and pathological stage, *COL1A2* remained associated with OS (n = 373; 146 events; HR 1.21, 95% CI 1.01-1.44; P = 0.036; **Figures 4A, C**). In Model B, which instead adjusted for T, N, and M separately, the estimate was attenuated (n = 360; 142 events; HR 1.14, 95% CI 0.95-1.37; P = 0.171; **Figures 4B, C**). Global proportional-hazards tests were nonsignificant for Model A (P = 0.840) and Model B (P = 0.189). Adding *COL1A2* increased the median bootstrap C-index from 0.670 to 0.676 in Model A (difference 0.006, 95% CI -0.003 to 0.024) and from 0.682 to 0.685 in Model B (difference 0.003, 95% CI -0.004 to 0.017; **Figure 4D**). *COL1A2* therefore added little discrimination to either clinical model.

**Figure 4:**
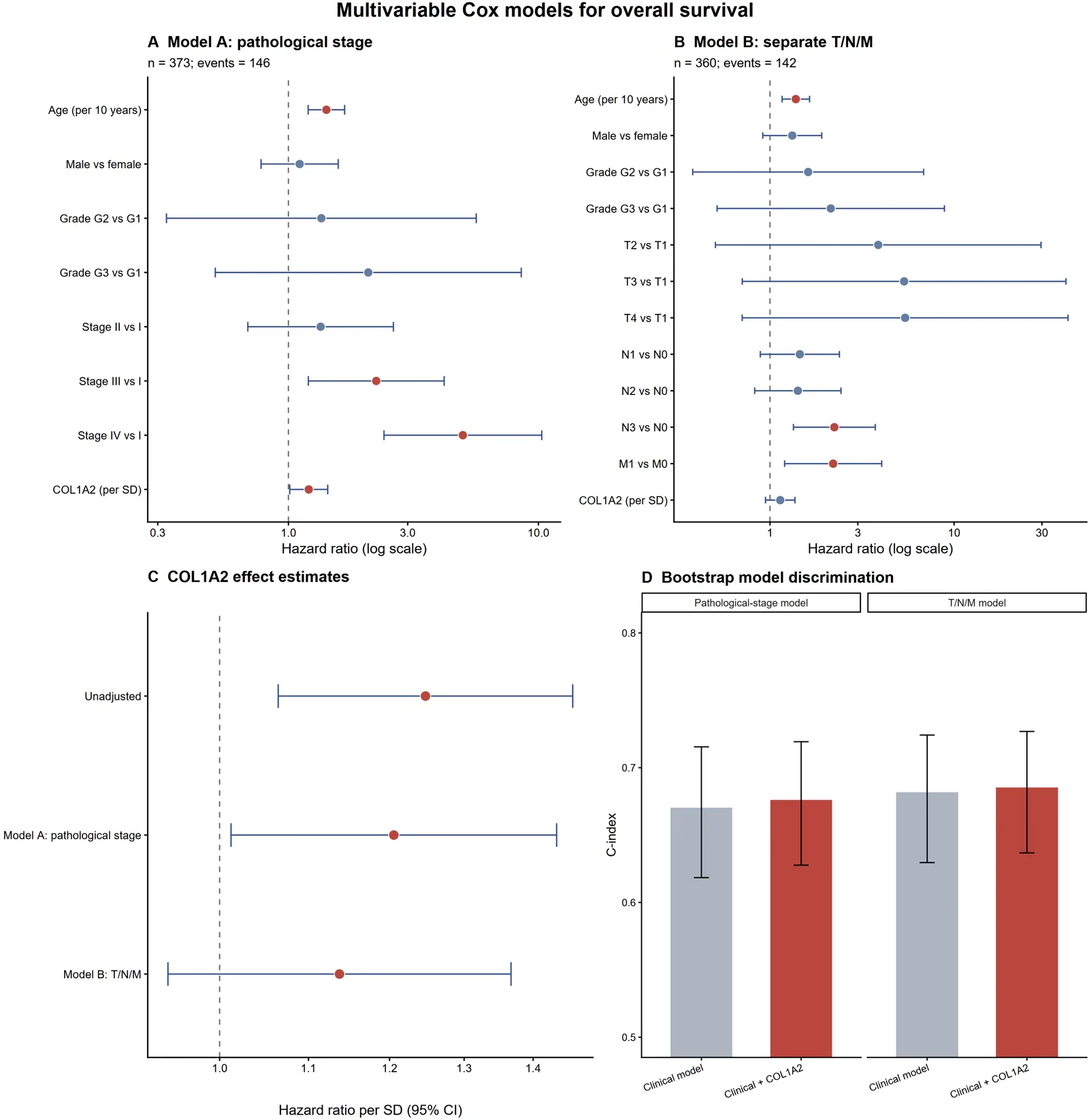
Multivariable Cox models for overall survival. **(A)** Model containing pathological stage but not T/N/M. **(B)** Model containing separate T/N/M classifications but not pathological stage. **(C)** *COL1A2* hazard ratios per standard-deviation increase in the unadjusted, pathological-stage, and T/N/M models. **(D)** Bootstrap Harrell C-indices for each clinical model before and after adding *COL1A2*. Error bars show 95% bootstrap intervals.

### 3.4 *COL1A2*-associated functional enrichment

Samples were divided into median-defined high- and low-COL1A2 groups, and differentially expressed genes (DEGs) were visualized in a volcano plot (**Figure 5A**). GO and KEGG analyses associated these DEGs with ECM and extracellular structure organization, ossification, collagen-containing ECM, focal adhesion, PI3K-Akt signaling, and related pathways (**Figures 5B, C**). GSEA also identified enrichment of cell adhesion molecule, ECM-receptor interaction, focal adhesion, olfactory transduction, and cancer-related gene sets (**Figure 5D**). The predominant enrichment pattern involved ECM organization and cell-matrix interactions, consistent with a stromal transcriptional phenotype rather than proof of tumor-cell-autonomous effects.

**Figure 5:**
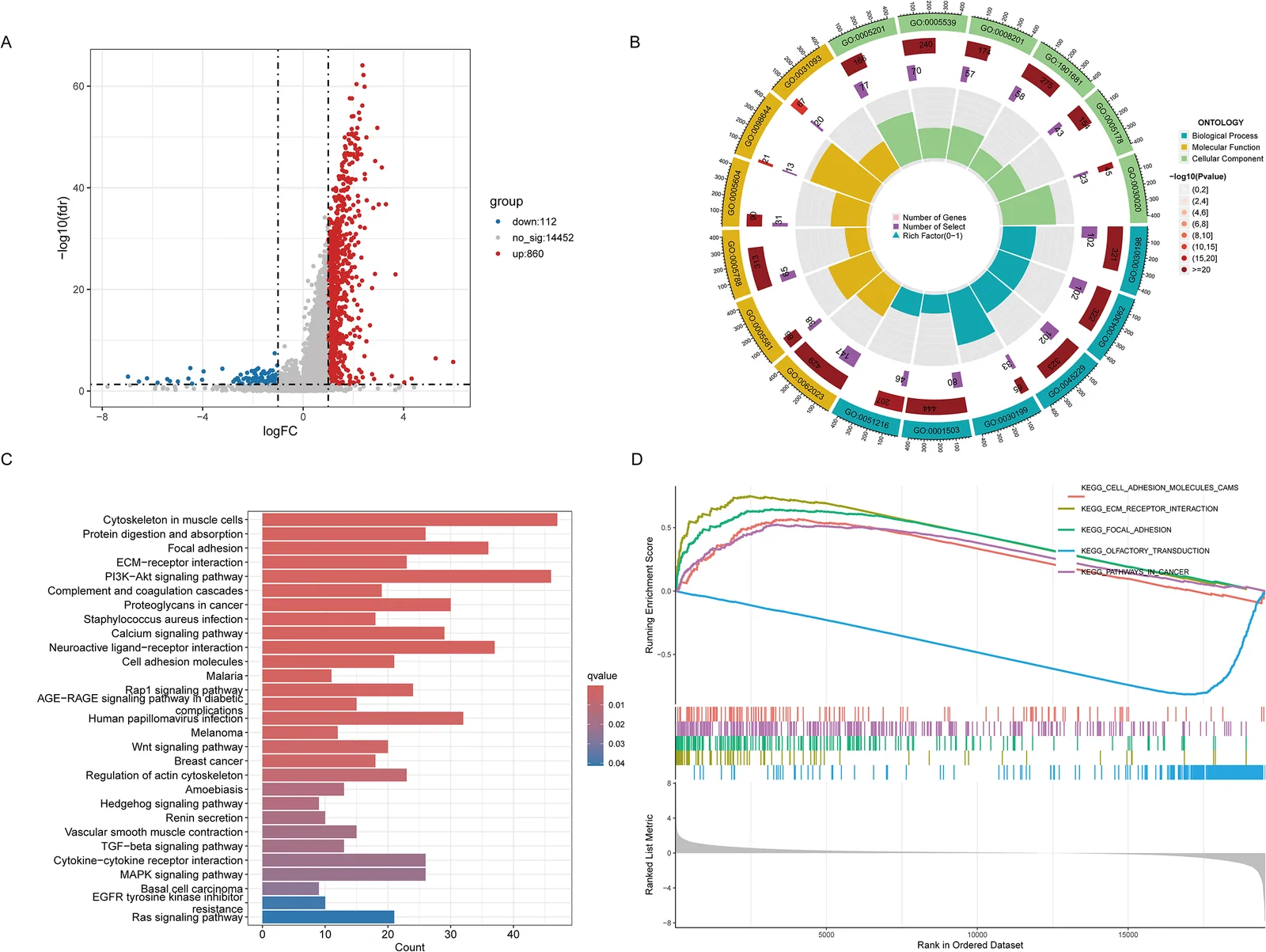
Functional enrichment associated with *COL1A2* expression. **(A)** Volcano plot of DEGs between the high- and low-*COL1A2* groups. **(B)** GO enrichment circle plot. **(C)** KEGG pathway enrichment plot. **(D)** GSEA enrichment curves.

### 3.5 Immune infiltration and immunotherapy response scores

ESTIMATE analysis showed higher stromal, immune, and composite ESTIMATE scores in the median-defined high-COL1A2 group (**Figure 6A**). CIBERSORT estimates showed greater M2 macrophage abundance in the high-expression group and greater plasma-cell and regulatory T-cell abundance in the low-expression group (**Figures 6B, C**). COL1A2 expression also correlated with several immune-checkpoint transcripts (**Figure 6D**).

**Figure 6:**
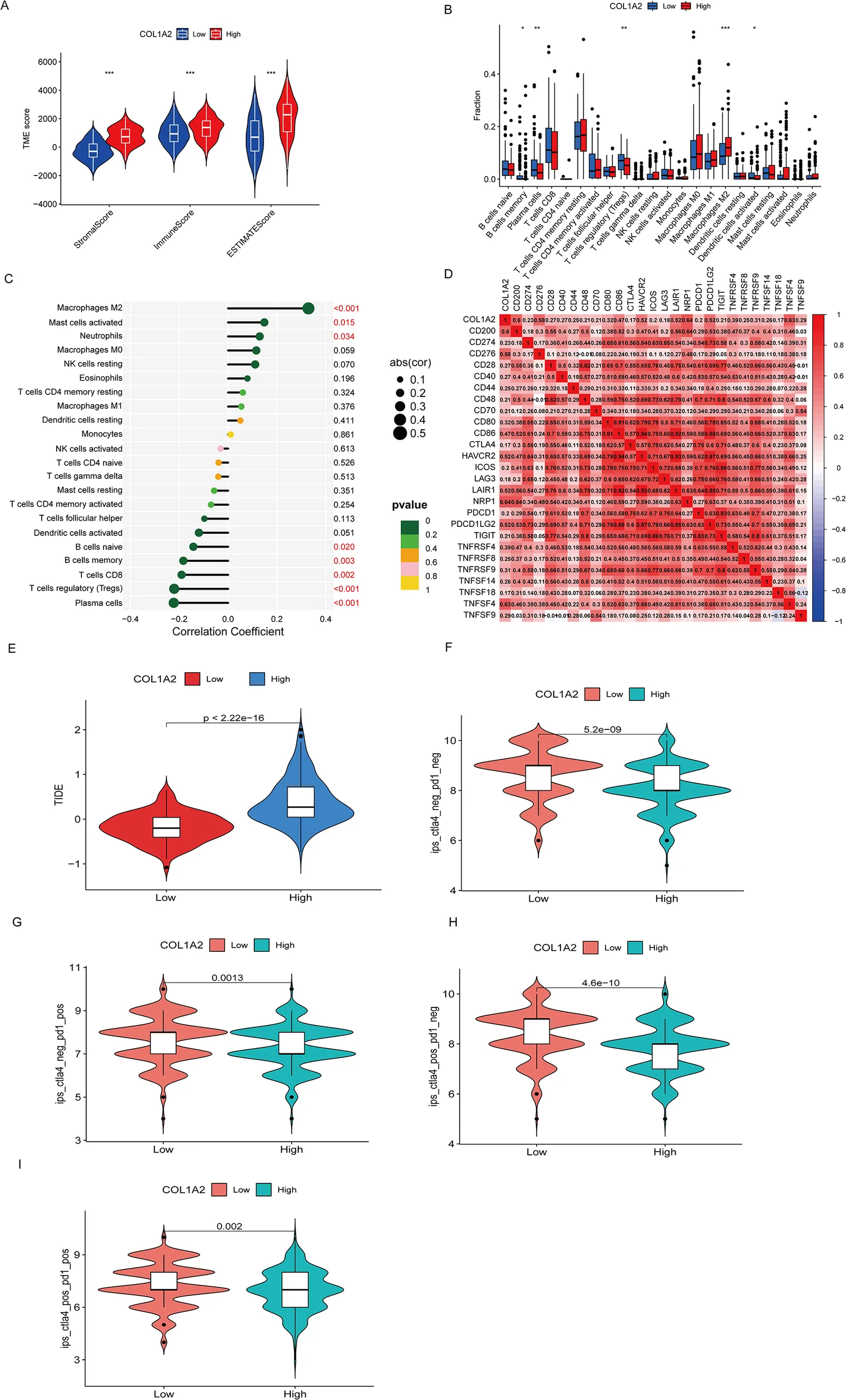
Immune infiltration estimates and immunotherapy response scores by *COL1A2* expression. **(A)** Stromal, immune, and ESTIMATE scores. **(B)** CIBERSORT-estimated immune-cell fractions. **(C)** Correlations between *COL1A2* expression and estimated immune-cell abundance. **(D)** Correlations with immune-checkpoint transcripts. **(E)** TIDE scores for the high and low expression groups of COL1A2. **(F)** CTLA4-negative/PD-1-negative, **(G)** CTLA4-negative/PD-1-positive, **(H)** CTLA4-positive/PD-1-negative, and **(I)** CTLA4-positive/PD-1-positive strata.

The low-COL1A2 group had lower TIDE values than the high-expression group (**Figure 6E**). Across the four CTLA4/PD-1 IPS strata, the low-expression group also had higher IPS values (**Figures 6F–I**). Both scoring systems therefore indicated a less favorable model-based ICI response score in the high-COL1A2 group.

### 3.6 Identification of genes associated with immunotherapy response scores

WGCNA was used to identify modules associated with the IPS and TIDE scores. The blue module showed the strongest absolute module-trait correlations and was selected for downstream analysis (**Figure 7A**). GO and KEGG analyses associated its genes with ECM organization, collagen metabolism, skeletal development, immune-related processes, and the tumor microenvironment (**Supplementary Figure 2**). A STRING PPI network was visualized in Cytoscape, and MCODE identified 18 densely connected candidates: *COL6A3*, *ELN*, *FN1*, *COL5A2*, *SPARC*, *LUM*, *BGN*, *COL5A3*, *COL6A1*, *COL12A1*, *COL16A1*, *MMP2*, *COL3A1*, *THBS2*, *DCN*, *COL6A2*, *COL11A1*, and *COL5A1* (**Figure 7B**).

**Figure 7:**
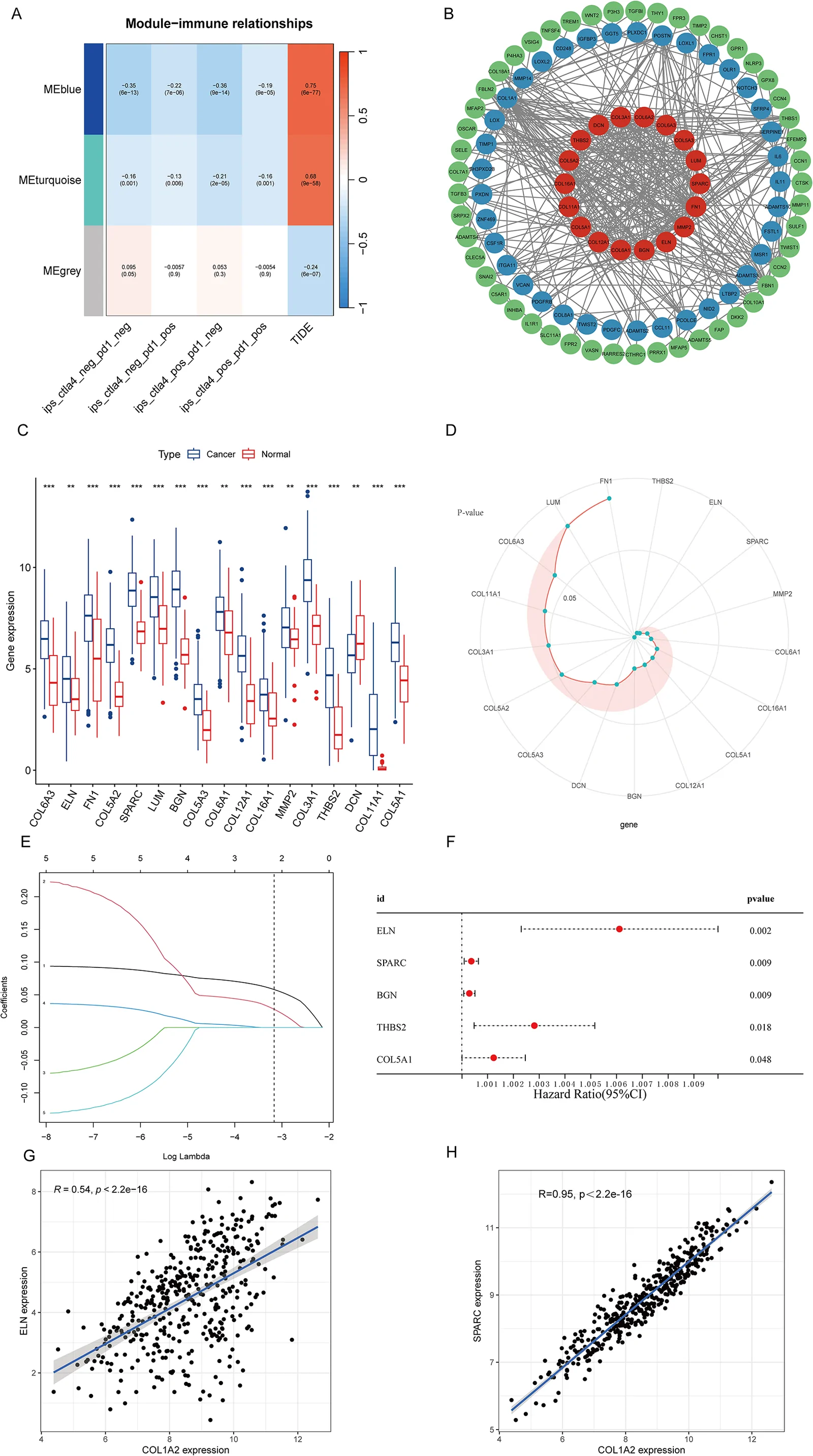
Identification of COL1A2-associated network genes. **(A)** WGCNA module correlations with IPS strata and TIDE. **(B)** PPI network and MCODE candidates. **(C)** Candidate-gene expression in normal and GC tissues. **(D)** Radar plot of Kaplan-Meier P values. **(E)** LASSO coefficient paths for COL1A2 and five survival-screened ECM-related genes. **(F)** Univariate Cox forest plot. **(G, H)** Bulk-tissue correlations of COL1A2 with ELN and SPARC, respectively. Ten-fold lambda tuning and 200-repeat selection stability are provided in **Supplementary Figure 3**.

Expression comparisons for the network genes are shown in **Figure 7C**, and Kaplan-Meier screening results are summarized in **Figure 7D**. Univariate Cox estimates for SPARC, THBS2, ELN, BGN, and COL5A1 are shown in **Figures 7E**, **F**. When COL1A2 was included with these five genes, all six genes had non-zero coefficients at lambda.min, whereas no gene was retained at lambda.1se. Across 200 repeated ten-fold cross-validation runs, SPARC and ELN were selected in 100% of lambda.min models and BGN in 98.5% (**Figures 7G**, **H**); THBS2, COL5A1, and COL1A2 were selected in 6.5%, 7.5%, and 5.5% of runs, respectively (**Supplementary Figure 3**). These results identify a reproducible ECM-related gene set in the TCGA-STAD cohort.

### 3.7 Validation of *COL1A2* mRNA and COL1A2 protein abundance in paired GC tissues

COL1A2 mRNA and COL1A2 protein abundance were assessed in seven paired GC and adjacent non-tumor tissues. qPCR showed higher COL1A2 mRNA abundance in tumor tissues than in matched adjacent tissues (mean relative expression, 1.83 +/- 0.28 vs 1.01 +/- 0.01; paired P < 0.001; **Figure 1A**), and Western blotting showed higher normalized COL1A2 protein abundance (mean COL1A2/beta-actin ratio, 1.20 +/- 0.27 vs 0.73 +/- 0.16; paired P < 0.001; **Figures 1B, C**). Together, these RNA and protein assays support increased bulk-tissue COL1A2 abundance in this small paired series, but they do not define cellular localization. The paired quantitative values underlying **Figure 7** are provided in **Supplementary Figure 2**.

## 4 Discussion

GC remains a major cause of cancer mortality, and reliable biomarkers are needed to improve prognostic stratification. COL1A2 was more highly expressed in tumor tissues and was associated with poorer OS in TCGA-STAD, but its 1-, 3-, and 5-year AUC values of 0.60, 0.65, and 0.66 indicate modest discrimination. In the multivariable analyses, COL1A2 remained associated with OS in the pathological-stage model (HR 1.21, P = 0.036) but not in the separate T/N/M model (HR 1.14, P = 0.171). Adding COL1A2 improved the bootstrap C-indices by only 0.006 and 0.003, with confidence intervals that included zero. In the independent ACRG GSE62254 cohort, the univariable association was not statistically significant (HR 1.83, P = 0.085; log-rank P = 0.120). These results support an outcome association in the discovery cohort, not a robust or independently validated standalone prognostic biomarker.

COL1A2 encodes the alpha2 chain of type I collagen and is closely related to the collagen-rich ECM that is prominent in GC. Functional analyses associated high COL1A2 transcript abundance with ECM organization, ECM-receptor interactions, focal adhesion, and related pathways. These findings are consistent with the biological role of type I collagen but also point to the importance of cellular origin. In GC, collagen-rich ECM is commonly produced by cancer-associated fibroblasts and other stromal cells (23). Bulk-tissue COL1A2 transcript abundance may therefore reflect fibroblast abundance or reactive stromal remodeling rather than a tumor-cell-autonomous program. The qPCR assay confirmed higher COL1A2 mRNA abundance, whereas Western blotting confirmed higher tissue-level COL1A2 protein abundance; neither assay distinguished stromal from epithelial expression.

CIBERSORT estimates associated high *COL1A2* expression with greater M2 macrophage infiltration and lower plasma-cell and Treg abundance. M2 macrophages can contribute to GC microenvironment remodeling and metastatic phenotypes (24, 25). The lower Treg estimate, together with higher stromal and ESTIMATE scores, may reflect broader differences in tissue composition or immune exclusion rather than selective Treg recruitment. TIDE and IPS showed a concordant pattern, with less favorable model-based ICI response scores in the high-*COL1A2* group. These immune associations are consistent with a stroma-rich, immune-excluded tumor phenotype.

The WGCNA, PPI, survival-screening, Cox, and LASSO workflow identified several ECM-related genes. After inclusion of COL1A2 in the penalized model, SPARC and ELN were selected in every repeated lambda.min analysis and BGN in 98.5%, whereas COL1A2 was selected in 5.5% of runs; no gene was retained under lambda.1se. ELN has been associated with epithelial-mesenchymal transition and vimentin-positive fibroblasts in GC (26), while SPARC can modulate extracellular signaling and angiogenesis (27, 28). The correlations among COL1A2, SPARC, ELN, and BGN may reflect a shared fibroblast-rich stromal program, although their regulatory relationships remain unresolved.

Important limitations should be noted. Cellular origin cannot be assigned from bulk expression, the adjusted prognostic estimate varied between clinical models, and the increase in C-index was small. GSE63089 independently confirmed the tumor-versus-adjacent-tissue expression difference but did not contain survival data and therefore did not validate the prognostic association. GSE62254 provided 300 gastric-tumor expression profiles with matched OS records; 152 observations were deaths and 148 were censored. In this external cohort, COL1A2 was not significantly associated with OS in univariable Cox analysis (HR 1.83, 95% CI 1.02-3.30; P = 0.085; log-rank P = 0.120). This external analysis did not include covariate adjustment because the expression matrix and the clinical table did not provide a harmonized set of covariates for the matched analysis. TIDE and IPS were not evaluated against outcomes in an independent ICI-treated GC cohort or compared with MSI, EBV, PD-L1 combined positive score, and tumor mutational burden. The LASSO candidates were assessed internally without spatial or functional validation. The seven-pair tissue series established the direction of expression change but was not intended for immune-marker assessment, subgroup analysis, or mechanistic testing. The laboratory reports provide the qPCR primer sequences, 146-bp COL1A2 amplicon, approximate 60-degree-C annealing temperature, and technical-replicate number. They also provide Western blot antibody manufacturers, catalog numbers, dilutions, and the supplied host/clonality information. Original IHC images, scale calibration, and scoring records could not be retrieved, so IHC was not used as validation evidence. Individual clinicopathological information, standardized tumor-margin distance, and independent pathological confirmation of adjacent-tissue status were also not retained, preventing retrospective reconstruction of a clinicopathological table.

## 5 Conclusions

COL1A2 expression was elevated in GC and was associated with poorer survival in TCGA-STAD, a stroma-rich microenvironment, M2 macrophage infiltration, and less favorable model-based immunotherapy response scores. Its prognostic contribution beyond clinicopathological variables was small and varied between model specifications, and the association was not significant in the external ACRG GSE62254 cohort. SPARC, ELN, and BGN showed high selection stability in the exploratory LASSO analysis. Taken together, the data identify COL1A2 as a marker associated with an adverse stromal phenotype in GC and provide a rationale for future cell-resolved, external-validation, and functional studies; they do not establish COL1A2 as a clinically validated prognostic biomarker or therapeutic target.

## Data Availability

The data that support the findings of this study are openly available in TCGA database at https://www.cancer.gov/ccg/research/genome-sequencing/tcga, GEO database at https://www.ncbi.nlm.nih.gov/geo and TIMER 2.0 at http://timer.cistrome.org/. The data in the article has been uploaded to the FigShare database.The data link https://doi.org/10.6084/m9.figshare.34746945. The public database data used in this article are all publicly accessible. The experimental data generated in this study are all shared in the MENDELEY database in an open state and can be viewed at any time. The data link is https://doi.org/10.17632/y65wkfdkcr.1.

## Supplementary material

Supplementary Figure 1 External overall-survival validation of COL1A2 in the ACRG GSE62254 cohort. Kaplan-Meier curves compare median-defined COL1A2 expression groups in 300 matched gastric-tumor profiles (152 deaths and 148 censored observations; log-rank P = 0.120).

Supplementary Figure 2 Functional enrichment analysis of differentially expressed genes. **(A)** GO enrichment analysis. The grouped bar chart shows significantly enriched Gene Ontology terms in the biological process (BP), cellular component (CC), and molecular function (MF) categories. The x-axis shows the number of genes, and color represents the P value. Enriched terms predominantly involve ECM organization, collagen-containing structures, and binding functions. **(B)** KEGG enrichment analysis. The bar chart shows significantly enriched KEGG pathways; the x-axis shows the number of genes and color represents the P value. Prominent pathways include ECM-receptor interaction, protein digestion and absorption, and PI3K-Akt signaling.

Supplementary Figure 3 LASSO lambda tuning and selection stability for COL1A2 and five ECM-related genes. **(A)** Ten-fold cross-validation curve based on partial-likelihood deviance; dashed lines identify lambda.min and lambda.1se. **(B)** Gene-selection frequencies across 200 repeated ten-fold cross-validation runs under the lambda.min and lambda.1se rules. At lambda.min, SPARC and ELN were selected in 100% of runs, BGN in 98.5%, COL5A1 in 7.5%, THBS2 in 6.5%, and COL1A2 in 5.5%; lambda.1se selected no genes.

Supplementary Table 1 Available de-identified characteristics of the seven-pair validation cohort.

Supplementary Table 2 De-identified paired qPCR and Western blot quantitative values underlying **Figure 1**.

## References

[1] Sung H Ferlay J Siegel RL Laversanne M Soerjomataram I Jemal A . Global cancer statistics 2020: GLOBOCAN estimates of incidence and mortality worldwide for 36 cancers in 185 countries. CA Cancer J Clin. (2021) 71:209–49. doi: 10.3322/caac.21660 33538338

[2] Cenitagoya GF Bergh CK Klinger-Roitman JA . A prospective study of gastric cancer. “Real” 5-year survival rates and mortality rates in a country with high incidence. Dig Surg. (1998) 15:317–22. doi: 10.1159/000018645 9845606

[3] Song Z Wu Y Yang J Yang D Fang X . Progress in the treatment of advanced gastric cancer. Tumour Biol. (2017) 39:1010428317714626. doi: 10.1177/1010428317714626 28671042

[4] Wagner AD Syn NL Moehler M Grothe W Yong WP Tai B-C . Chemotherapy for advanced gastric cancer. Cochrane Database Syst Rev. (2017) 8:CD004064. doi: 10.1002/14651858.CD004064.pub4 28850174 PMC6483552

[5] Mouw JK Ou G Weaver VM . Extracellular matrix assembly: A multiscale deconstruction. Nat Rev Mol Cell Biol. (2014) 15:771–85. doi: 10.1038/nrm3902 25370693 PMC4682873

[6] Cox TR Erler JT . Remodeling and homeostasis of the extracellular matrix: Implications for fibrotic diseases and cancer. Dis Model Mech. (2011) 4:165–78. doi: 10.1242/dmm.004077 21324931 PMC3046088

[7] Majo S Auguste P . The yin and yang of discoidin domain receptors (DDRs): Implications in tumor growth and metastasis development. Cancers (Basel). (2021) 13:1725. doi: 10.3390/cancers13071725 33917302 PMC8038660

[8] Ricard-Blum S . The collagen family. Cold Spring Harb Perspect Biol. (2011) 3:a004978. doi: 10.1101/cshperspect.a004978 21421911 PMC3003457

[9] Walker C Mojares E Del Río Hernández A . Role of extracellular matrix in development and cancer progression. Int J Mol Sci. (2018) 19:3028. doi: 10.3390/ijms19103028 30287763 PMC6213383

[10] Xu S Xu H Wang W Li S Li H Li T . The role of collagen in cancer: From bench to bedside. J Transl Med. (2019) 17:309. doi: 10.1186/s12967-019-2058-1 31521169 PMC6744664

[11] Guszczyn T Sobolewski K . Deregulation of collagen metabolism in human stomach cancer. Pathobiology. (2004) 71:308–13. doi: 10.1159/000081726 15627841

[12] Jang M Koh I Lee JE Lim JY Cheong J-H Kim P . Increased extracellular matrix density disrupts E-cadherin/β-catenin complex in gastric cancer cells. Biomater Sci. (2018) 6:2704–13. doi: 10.1039/c8bm00843d 30151505

[13] Zhu Y Liu W Chen S Xu F Zhang L Hayashi T . Collagen type I enhances cell growth and insulin biosynthesis in rat pancreatic cells. J Mol Endocrinol. (2021) 67:135–48. doi: 10.1530/JME-21-0032 34370695

[14] Sengupta PK Fargo J Smith BD . The RFX family interacts at the collagen (*COL1A2*) start site and represses transcription. J Biol Chem. (2002) 277:24926–37. doi: 10.1074/jbc.M111712200 11986307

[15] Sadler T Scarpa M Rieder F West G Stylianou E . Cytokine-induced chromatin modifications of the type I collagen alpha 2 gene during intestinal endothelial-to-mesenchymal transition. Inflammation Bowel Dis. (2013) 19:1354–64. doi: 10.1097/mib.0b013e318281f37a 23635716 PMC3684204

[16] Dong Z Lin W Kujawa SA Wu S Wang C . Predicting microRNA target genes and identifying hub genes in IIA stage colon cancer patients using bioinformatics analysis. BioMed Res Int. (2019) 2019:6341967. doi: 10.1155/2019/6341967 30881993 PMC6383401

[17] Wang Y Sakaguchi M Sabit H Tamai S Ichinose T Tanaka S . *COL1A2* inhibition suppresses glioblastoma cell proliferation and invasion. J Neurosurg. (2023) 138:639–48. doi: 10.3171/2022.6.JNS22319 35932265

[18] Wu J Liu J Wei X Yu Q Niu X Tang S . A feature-based analysis identifies *COL1A2* as a regulator in pancreatic cancer. J Enzyme Inhib Med Chem. (2019) 34:420–8. doi: 10.1080/14756366.2018.1484734 30734598 PMC6327995

[19] Sengupta PK Smith EM Kim K Murnane MJ Smith BD . DNA hypermethylation near the transcription start site of collagen alpha2(I) gene occurs in both cancer cell lines and primary colorectal cancers. Cancer Res. (2003) 63:1789–97. 12702564

[20] Mori K Enokida H Kagara I Kawakami K Chiyomaru T Tatarano S . CpG hypermethylation of collagen type I α 2 contributes to proliferation and migration activity of human bladder cancer. Int J Oncol. (2009) 34(6):1593–602. doi: 10.3892/ijo_00000289 19424577

[21] Lim A Greenspan DS Smith BD . Expression of α2 type I collagen in W8 cells increases cell adhesion and decreases colony formation in soft agar. Matrix Biol. (1994) 14:21–30. doi: 10.1016/0945-053x(94)90026-4 8061917

[22] Cristescu R Lee J Nebozhyn M Kim KM Ting JC Wong SS . Molecular analysis of gastric cancer identifies subtypes associated with distinct clinical outcomes. Nat Med. (2015) 21:449–56. doi: 10.1038/nm.3850 25894828

[23] Moreira AM Pereira J Melo S Fernandes MS Carneiro P Seruca R . The extracellular matrix: An accomplice in gastric cancer development and progression. Cells. (2020) 9:394. doi: 10.3390/cells9020394 32046329 PMC7072625

[24] Zhao S Mi Y Guan B Zheng B Wei P Gu Y . Tumor-derived exosomal miR-934 induces macrophage M2 polarization to promote liver metastasis of colorectal cancer. J Hematol Oncol. (2020) 13:156. doi: 10.1186/s13045-020-00991-2 33213490 PMC7678301

[25] Li W Zhang X Wu F Zhou Y Bao Z Li H . Gastric cancer-derived mesenchymal stromal cells trigger M2 macrophage polarization that promotes metastasis and EMT in gastric cancer. Cell Death Dis. (2019) 10:918. doi: 10.1038/s41419-019-2131-y 31801938 PMC6892854

[26] Fang T Zhang L Yin X Wang Y Zhang X Bian X . The prognostic marker elastin correlates with epithelial-mesenchymal transition and vimentin-positive fibroblasts in gastric cancer. J Pathol Clin Res. (2023) 9:56–72. doi: 10.1002/cjp2.298 36226731 PMC9732685

[27] Cydzik M Abdul-Wahid A Park S Bourdeau A Bowden K Prodeus A . Slow binding kinetics of secreted protein, acidic, rich in cysteine-VEGF interaction limit VEGF activation of VEGF receptor 2 and attenuate angiogenesis. FASEB J. (2015) 29:3493–505. doi: 10.1096/fj.15-271775 25921830

[28] Lopez MV Blanco P Viale DL Cafferata EG Carbone C Gould D . Data from expression of a suicidal gene under control of the human secreted protein acidic and rich in cysteine (*SPARC*) promoter in tumor or stromal cells led to the inhibition of tumor cell growth. (2023) 5(10):2503–11. doi: 10.1158/1535-7163.c.6531863.v1 PMC2747019 17041094

