# Supplementary material for: COL1A2 expression is associated with prognosis, stromal remodeling, and predicted immunotherapy response in gastric cancer
Source: Front Oncol. 2026 Oct 30;16:1912517. doi: 10.3389/fonc.2026.1912517 (PMC13759422; doi:10.3389/fonc.2026.1912517)
Supplement: Supplementary file 1 [file SupplementaryFile1.zip › Supplementary_Material.docx]

**Supplementary material**

COL1A2 Expression Is Associated with Prognosis, Stromal Remodeling, and Predicted Immunotherapy Response in Gastric Cancer

Supplementary Figures 1-3 and Supplementary Tables 1-2. Supplementary Data 1 is supplied separately as Supplementary_Data_1.zip.

Supplementary Figure 1. External overall-survival validation of COL1A2 in the ACRG GSE62254 cohort. Kaplan-Meier curves compare median-defined COL1A2 expression groups in 300 matched gastric-tumor profiles (152 deaths and 148 censored observations; log-rank P = 0.120). File: Supplementary_Figure_1.png.

Supplementary Figure 2. Functional enrichment of genes in the blue WGCNA module. File: Supplementary_Figure_2.png.

(A) GO enrichment analysis. The grouped bar chart shows significantly enriched Gene Ontology terms in the biological process (BP), cellular component (CC), and molecular function (MF) categories. The x-axis shows the number of genes, and color represents the P value. Enriched terms predominantly involve ECM organization, collagen-containing structures, and binding functions. (B) KEGG enrichment analysis. The bar chart shows significantly enriched KEGG pathways; the x-axis shows the number of genes and color represents the P value. Prominent pathways include ECM-receptor interaction, protein digestion and absorption, and PI3K-Akt signaling.

Supplementary Figure 3. LASSO lambda tuning and selection stability for COL1A2 and five ECM-related genes. (A) Ten-fold cross-validation curve based on partial-likelihood deviance; dashed lines identify lambda.min and lambda.1se. (B) Gene-selection frequencies across 200 repeated ten-fold cross-validation runs under the lambda.min and lambda.1se rules. At lambda.min, SPARC and ELN were selected in 100% of runs, BGN in 98.5%, COL5A1 in 7.5%, THBS2 in 6.5%, and COL1A2 in 5.5%; lambda.1se selected no genes. File: Supplementary_Figure_3.png.

Supplementary Table 1. Available de-identified characteristics of the seven-pair validation cohort. File: Supplementary_Table_1.csv. Cohort record IDs enumerate the source clinical record list. They are independent of the assay Pair IDs in Supplementary Table 2 and the lane labels in Figure 1; no row-by-row linkage between these two numbering systems is implied.

| **Cohort record ID** | **Age, years** | **Sex** |  | **Collection date** |
| --- | --- | --- | --- | --- |
| Record 1 | 82 | Female |  | 2025-03-24 |
| Record 2 | 67 | Male |  | 2025-03-24 |
| Record 3 | 76 | Male |  | 2025-03-27 |
| Record 4 | 71 | Male |  | 2025-03-31 |
| Record 5 | 84 | Male |  | 2025-04-03 |
| Record 6 | 76 | Male |  | 2025-04-03 |
| Record 7 | 57 | Male |  | 2025-04-03 |

Supplementary Table 2. De-identified paired qPCR and Western blot quantitative values underlying Figure 1. File: Supplementary_Table_2.csv.

| **Pair ID** | **qPCR adjacent tissue (2^-DeltaDeltaCt)** | **qPCR tumor tissue (2^-DeltaDeltaCt)** | **WB adjacent tissue (COL1A2/beta-actin)** | **WB tumor tissue (COL1A2/beta-actin)** |
| --- | --- | --- | --- | --- |
| Pair 1 | 1.001 | 1.929 | 0.94 | 1.61 |
| Pair 2 | 1.001 | 2.259 | 0.83 | 1.28 |
| Pair 3 | 1.002 | 1.680 | 0.77 | 1.34 |
| Pair 4 | 1.003 | 1.368 | 0.53 | 0.96 |
| Pair 5 | 1.002 | 1.769 | 0.68 | 0.91 |
| Pair 6 | 1.021 | 2.026 | 0.85 | 1.37 |
| Pair 7 | 1.022 | 1.764 | 0.52 | 0.95 |

Values reproduce the displayed precision of the manuscript table. Each Pair ID denotes one biological tumor-adjacent tissue pair; technical replicates were averaged within each biological specimen.

Supplementary Data 1. Supporting analysis datasets, result tables, and source scripts for the TCGA-STAD reanalysis, external ACRG GSE62254 survival analysis, and paired tissue measurements. File: Supplementary_Data_1.zip.
